# Supplementary material for: Folic acid supplementation ameliorates long-term lipid metabolism following intrauterine growth restriction
Source: PLoS One. 2026 Apr 8;21(4):e0346676. doi: 10.1371/journal.pone.0346676 (PMC13061216; doi:10.1371/journal.pone.0346676)
Supplement: S1 Fig — (PDF) [file pone.0346676.s001.pdf]

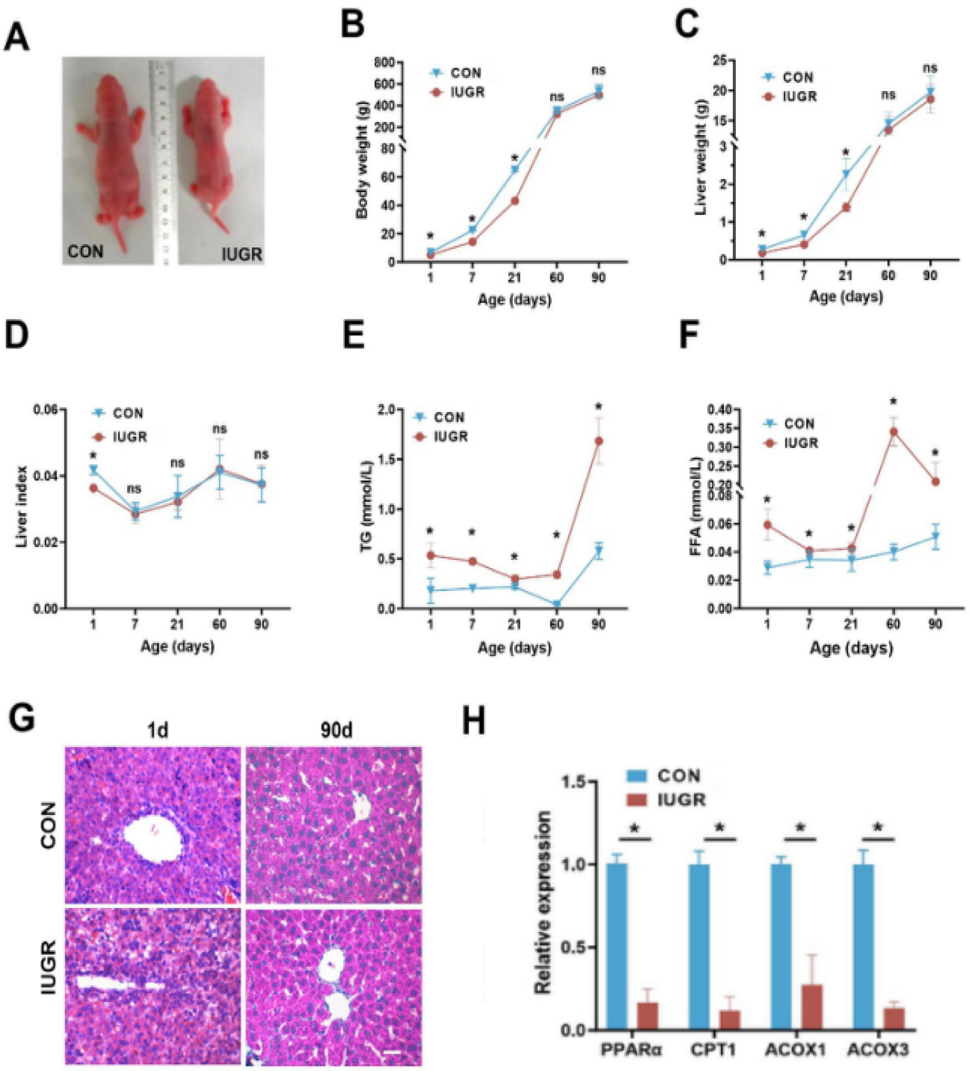

S1 Fig. IUGR induces lipid metabolism dysfunction that persists into adulthood in offspring.

(A) Representative images of newborn rats from the control group (left) and IUGR group (right). (B) Body weight trajectories of IUGR and control rats. (C-D) Liver weight and liver index trajectories of IUGR and control rats. (E-F) ELISA analysis of serum TG and FFA levels in IUGR and their control. (G) Representative H&E staining images of liver tissue from IUGR and control rats at 1 and 90 days after birth. (H) Expression levels of lipid metabolism-related genes in liver tissue were assessed by RT-PCR. CON: control; IUGR: Intrauterine Growth Restriction; TG: Triglycerides; FFA: Free Fatty Acids; PPAR: Peroxisome

Proliferator-Activated Receptor; ACOX: Acyl-CoA Oxidase; CPT: Carnitine Palmitoyltransferase. Statistics were performed using the student's t-test (B-F and H). *$p < 0.05$, ns: not significant.
