# Supplementary material for: Folic acid supplementation ameliorates long-term lipid metabolism following intrauterine growth restriction
Source: PLoS One. 2026 Apr 8;21(4):e0346676. doi: 10.1371/journal.pone.0346676 (PMC13061216; doi:10.1371/journal.pone.0346676)
Supplement: S2 Fig — (PDF) [file pone.0346676.s002.pdf]

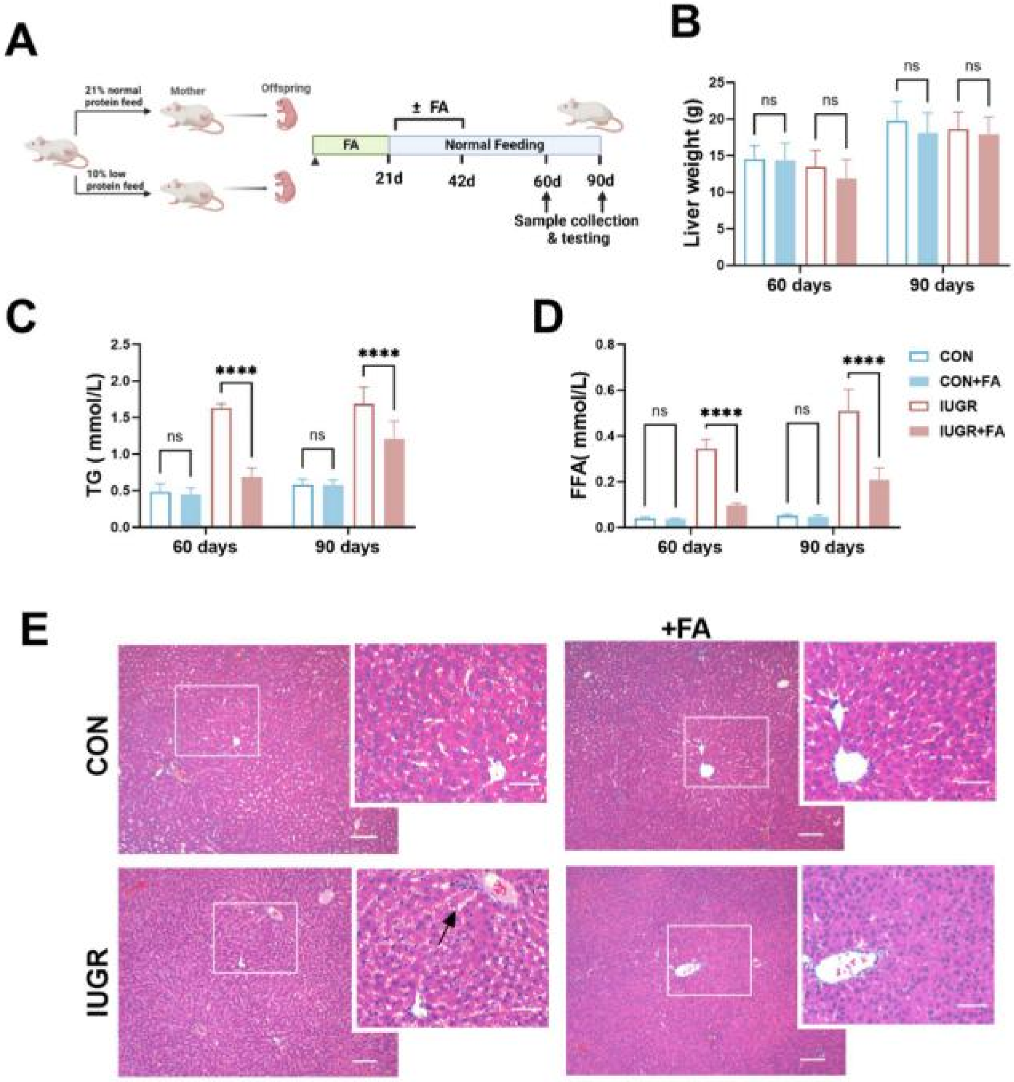

S2 Fig. The impact of early postnatal folic acid supplementation on IUGR-induced lipid metabolic dysfunction in adulthood

(A) Schematic representation of the experimental design. Both CON and IUGR rats were given a normal diet after weaning, and they received either a folic acid-supplemented treatment (CON+FA, IUGR+FA) or no supplementation for 3 weeks post-weaning. At 60 and 90 days of age, liver and serum samples were collected for further analysis. (B) Liver weights of rats in CON, CON+FA, IUGR and IUGR+FA group at 60 days and 90 days of age. (C-D) ELISA analysis of serum TG and FFA levels in these groups at 60 and 90 days. (E) Representative H&E staining images of liver tissue from IUGR and control rats at 1 and 90 days after birth.

CON: control; IUGR: Intrauterine Growth Restriction; FA: Folic acid; TG: Triglycerides; FFA: Free Fatty Acids. Statistics performed using student's t-test (B-D). ****$p < 0.0001$, ns: not significant.
