# Supplementary material for: Folic acid supplementation ameliorates long-term lipid metabolism following intrauterine growth restriction
Source: PLoS One. 2026 Apr 8;21(4):e0346676. doi: 10.1371/journal.pone.0346676 (PMC13061216; doi:10.1371/journal.pone.0346676)
Supplement: S3 Fig — (PDF) [file pone.0346676.s003.pdf]

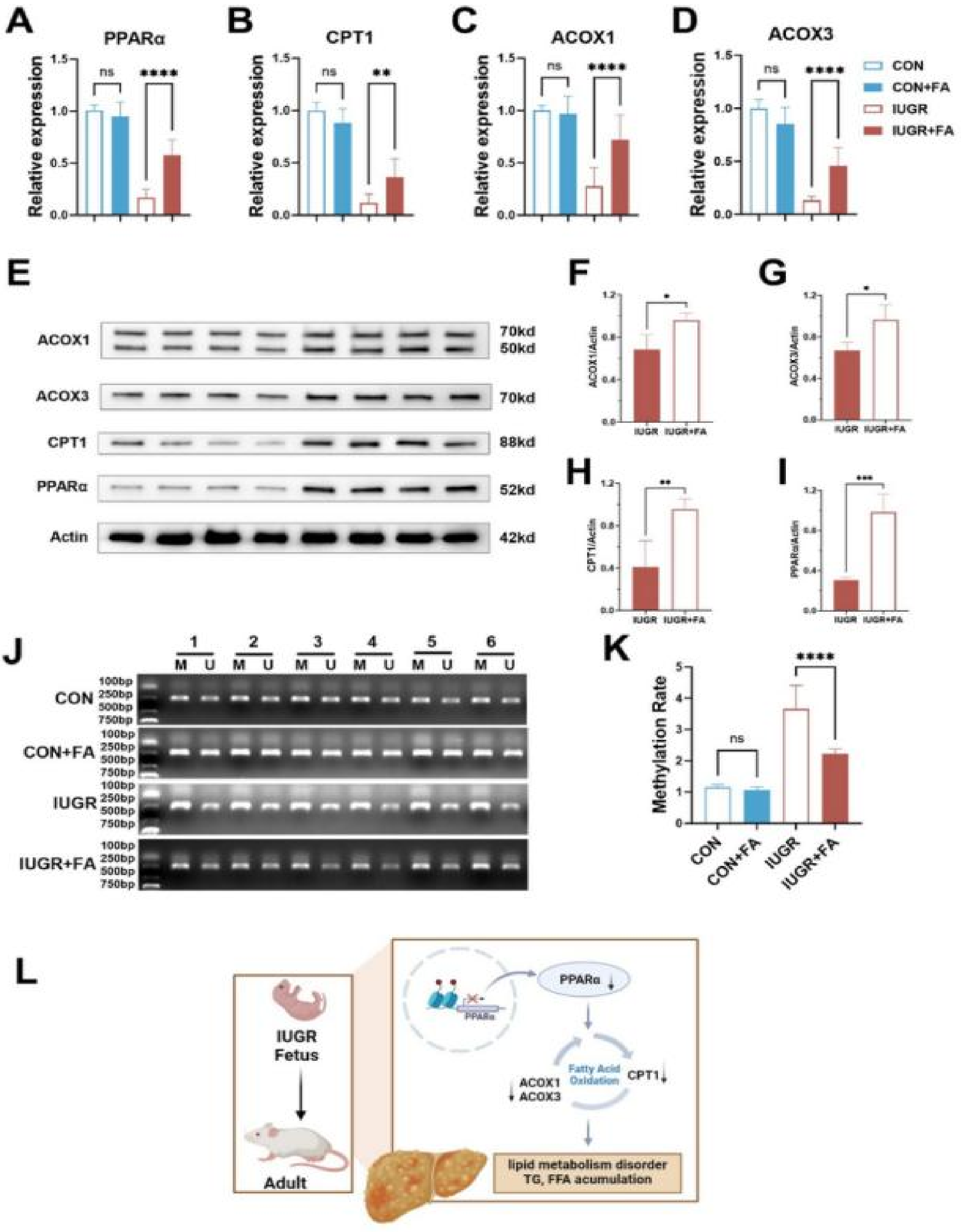

S3 Fig. Folic acid upregulates lipid-related genes in IUGR Rats

(A) Both CON and IUGR rats were given a normal diet after weaning, and they received either a folic acid-supplemented treatment (CON+FA, IUGR+FA) or no supplementation for 3 weeks post-weaning. At 90 days of age, liver samples were collected for further analysis. (A-D) RT-PCR showing the expression levels of PPARα (A), CPT1 (B), ACOX1 (C) and ACOX3 (D) in liver tissue from rats in CON, CON+FA, IUGR and

IUGR+FA group. (E) Representative image showing the protein levels of PPARα, CPT1, ACOX1 and ACOX3. (F-I) WB showing the levels of PPARα (F), CPT1 (G), ACOX1 (H) and ACOX3 (I) in liver tissue from rats in these groups. CON: control; IUGR: Intrauterine Growth Restriction; FA: Folic acid; PPAR: Peroxisome Proliferator-Activated Receptor; ACOX: Acyl-CoA Oxidase; CPT: Carnitine Palmitoyltransferase. Statistics performed using Student's t-test (A-D, F-I). *$p < 0.05$, **$p < 0.01$, ***$p < 0.001$, **$p < 0.0001$, ns: not significant.
