# Supplementary material for: Folic acid supplementation ameliorates long-term lipid metabolism following intrauterine growth restriction
Source: PLoS One. 2026 Apr 8;21(4):e0346676. doi: 10.1371/journal.pone.0346676 (PMC13061216; doi:10.1371/journal.pone.0346676)
Supplement: S1 Table — (PDF) [file pone.0346676.s004.pdf]

S1 Table. Composition and nutrient composition of experimental feed

	Corn (g/kg)	Third-grade flour (g/kg)	Wheat bran (g/kg)	Bean (g/kg)	Fish meal (g/kg)	Protein （%）	Fat （%）	Carbohydrates （%）
Protein-restricted diet	350	200	150	200	100	21	3.7	55
Normal diet	530	430	20	10	10	10	3.5	67
