# Supplementary figures and images for: Folic acid supplementation ameliorates long-term lipid metabolism following intrauterine growth restriction
Source: PLoS One. 2026 Apr 8;21(4):e0346676. doi: 10.1371/journal.pone.0346676 (PMC13061216; doi:10.1371/journal.pone.0346676)

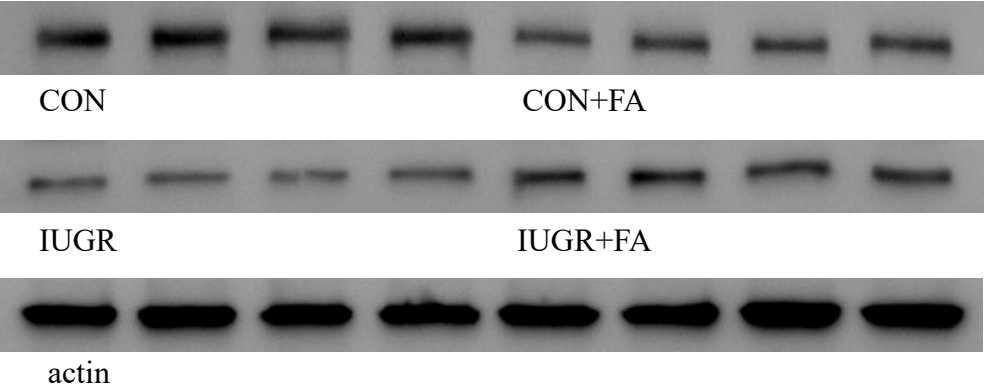

ACOX1

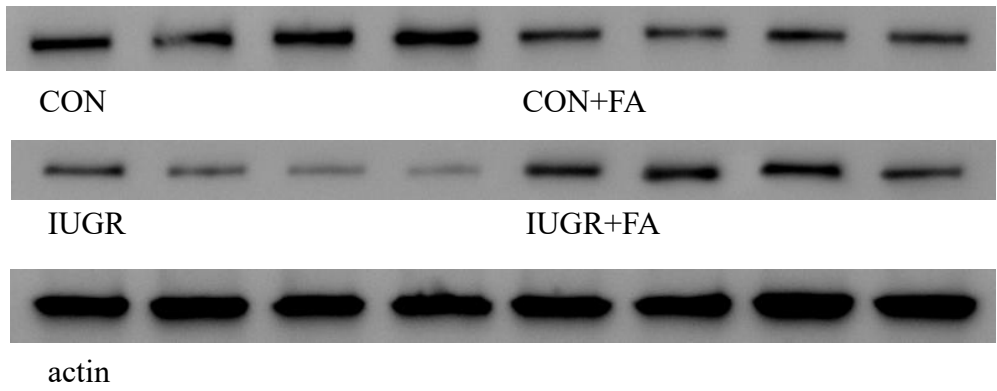

CPT1

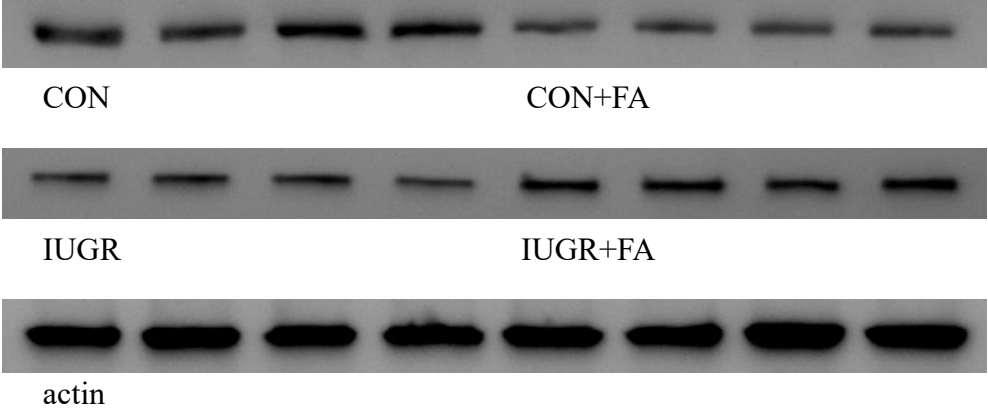

ACOX3

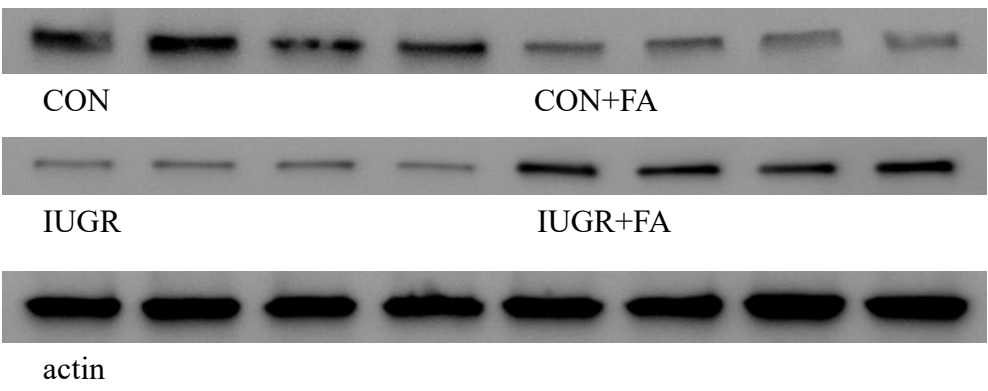

PPAR $\alpha$

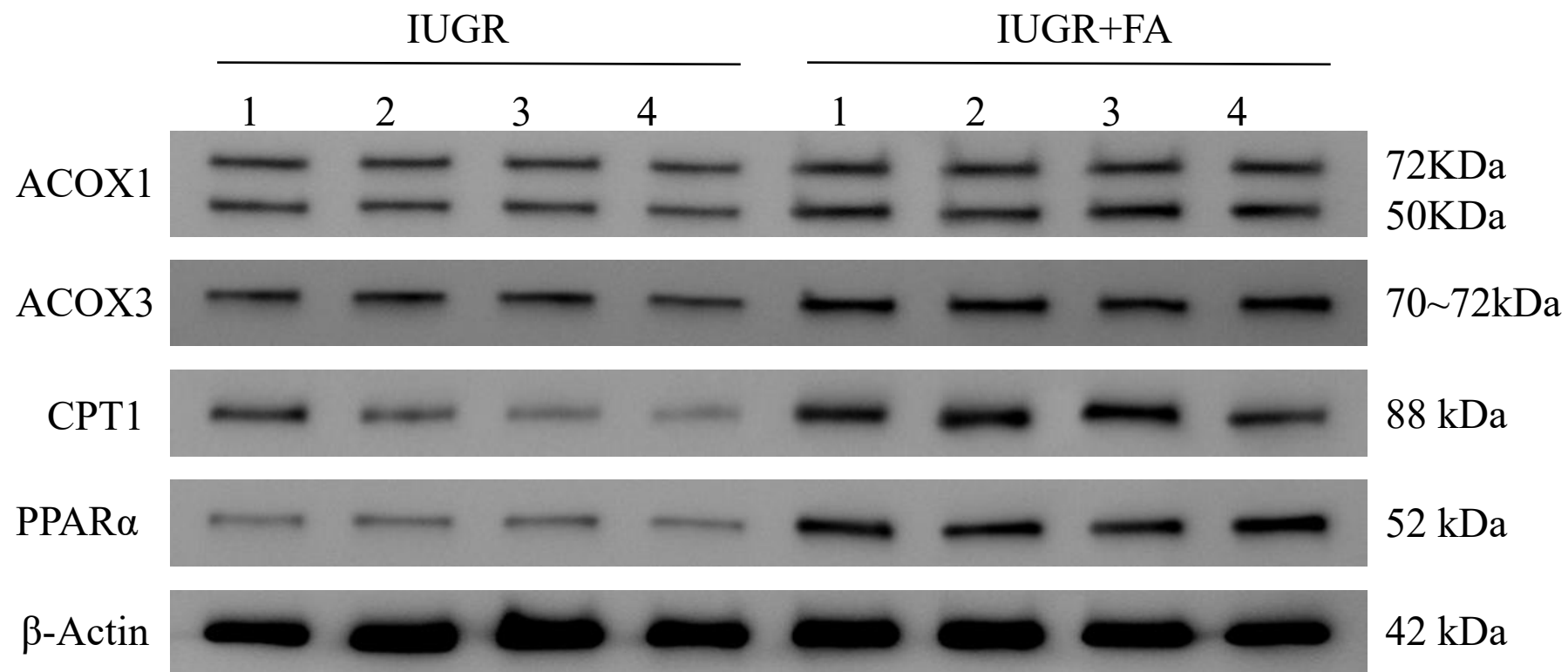

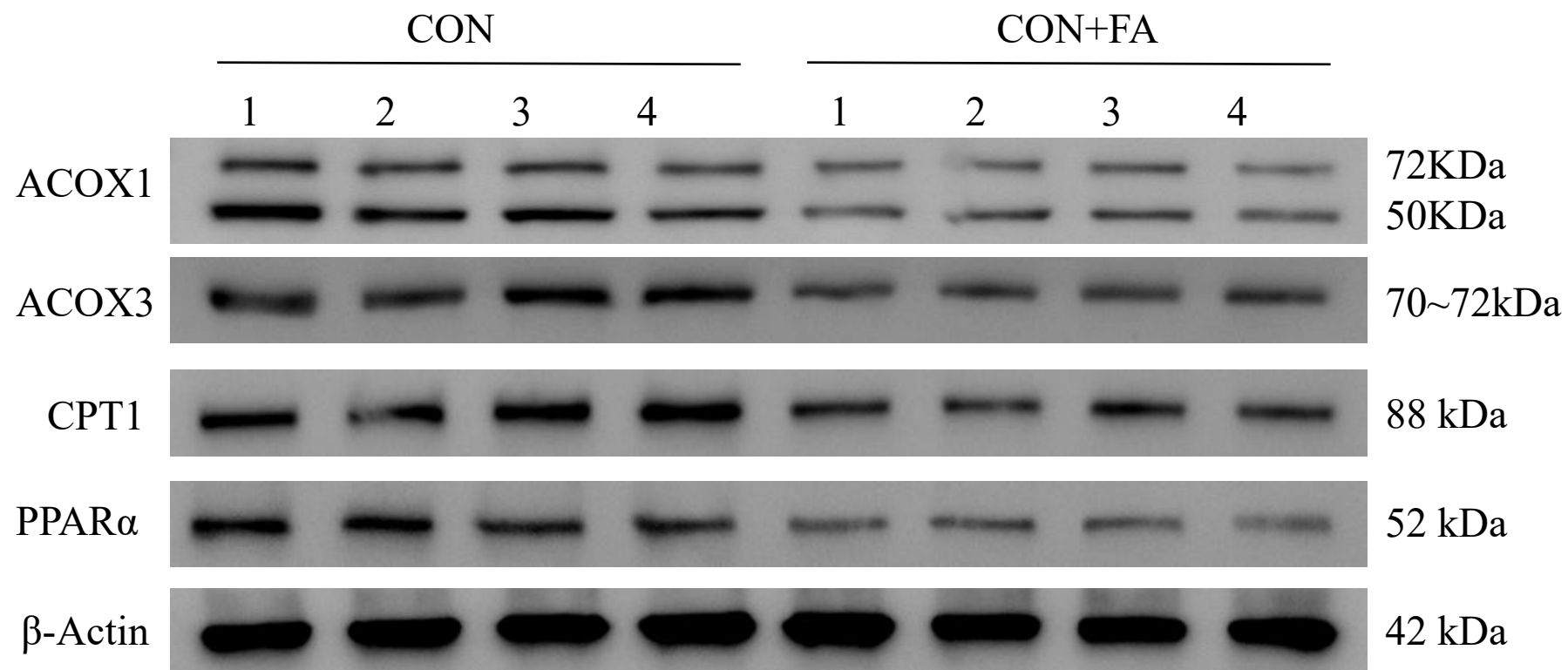

Supplement: S1 Data — (ZIP) [file pone.0346676.s005.zip › S1_raw_images/WB.pdf]
