# Supplementary material for: Folic acid supplementation ameliorates long-term lipid metabolism following intrauterine growth restriction
Source: PLoS One. 2026 Apr 8;21(4):e0346676. doi: 10.1371/journal.pone.0346676 (PMC13061216; doi:10.1371/journal.pone.0346676)
Supplement: Attachment — Submitted filename: PONE_D_25_63128 review.docx [file pone.0346676.s006.docx]

**Title: The effect of intrauterine growth retardation on long-term lipid metabolism in the liver and nutrition regulation**

This study provides substantial evidence, using an animal model, that intrauterine growth restriction (IUGR) leads to dysregulation of hepatic lipid metabolism in adulthood and that early postnatal folate supplementation exerts sustained beneficial effects on these metabolic disturbances. The work is well grounded in the DOHaD (Developmental Origins of Health and Disease) framework and demonstrates high clinical relevance as an early-life nutritional intervention strategy. The experimental design is sound, and the results are consistent and robust throughout the study.

- Methods
- P14	A total of 146 rat pups were included in this study; however, no sex-stratified analyses were presented. Given that responses may differ by sex, it would be important to include sex-specific analyses if such data are available.
- p14;14	Folic acid supplementation was initiated from postnatal day 21; could you please clarify the rationale for selecting this time point?

Could the authors clarify how the folic acid dosage was determined, and whether this dose corresponds to a standard physiological dose or a supraphysiological (high) dose?

- P17;1	Statistical analysis – The authors are encouraged to consider re-analyze longitudinal data using repeated-measures ANOVA or mixed-effects models to account for intra-individual correlation over time.
- Results
- The “Results” section currently includes descriptions that would be more appropriately placed in the “Methods” and/or “Discussion” sections. It would be necessary to revise this section so that it focuses exclusively on the presentation of experimental results.

Example) p18. We induced IUGR offspring models in offspring using maternal calorie and protein restriction, which has been previously validated. 🡪 This description would be more appropriate for the Methods section

P19. As reported, PPARα signals were essential for regulating TG deposition and hepatic damage for IUGR.

- Figures
- References to Figure 3J, 3K are missing from the main text.

Therefore, this manuscript represents a study with considerable merit for publication.

Thank you.
